# Air-Leak Syndrome with Spontaneous Tension Pneumothorax and Pneumomediastinum Caused by Bleomycin-Induced Organizing Pneumonia

**DOI:** 10.5334/jbsr.2880

**Published:** 2022-12-30

**Authors:** Cedric Vanmarcke, Thomas Steelandt, Anne-Sophie Vliegen

**Affiliations:** 1 UZ Leuven, BE; 2 Jessa Ziekenhuis, BE

**Keywords:** bleomycin, organizing pneumonia, air leak syndrome, Masson bodies, pneumothorax, pneumomediastinum, pneumatocele, bleb

## Abstract

**Teaching Point:** The appearance of pneumatoceles in a patient with organizing pneumonia is an early sign of leak syndrome and is a significant finding as its rupture can lead to a life-threatening tension pneumothorax.

Air leak syndrome (ALS), consisting of pneumothorax, subcutaneous emphysema and pneumomediastinum, is a rare complication of organizing pneumonia (OP). We report a case of a patient with bleomycin-induced OP who develops a tension pneumothorax due to a ruptured pneumatocele caused by ALS and correlate with histopathological analysis. This is the third case in the literature with these findings and the first with a tension pneumothorax.

## Introduction

Bleomycin is a common cause for drug-induced pulmonary toxicity, sometimes presenting as organizing pneumonia (OP) [1]. A rare complication of OP is pneumatocele formation with spontaneous pneumothorax and pneumomediastinum, the so-called ‘air-leak syndrome’ (ALS) [2,3]. We present the evolution in time using chest CT scans of a patient with bleomycin-induced OP complicated by ALS, as well as histopathological correlation.

## Case History

**Figure 1:**
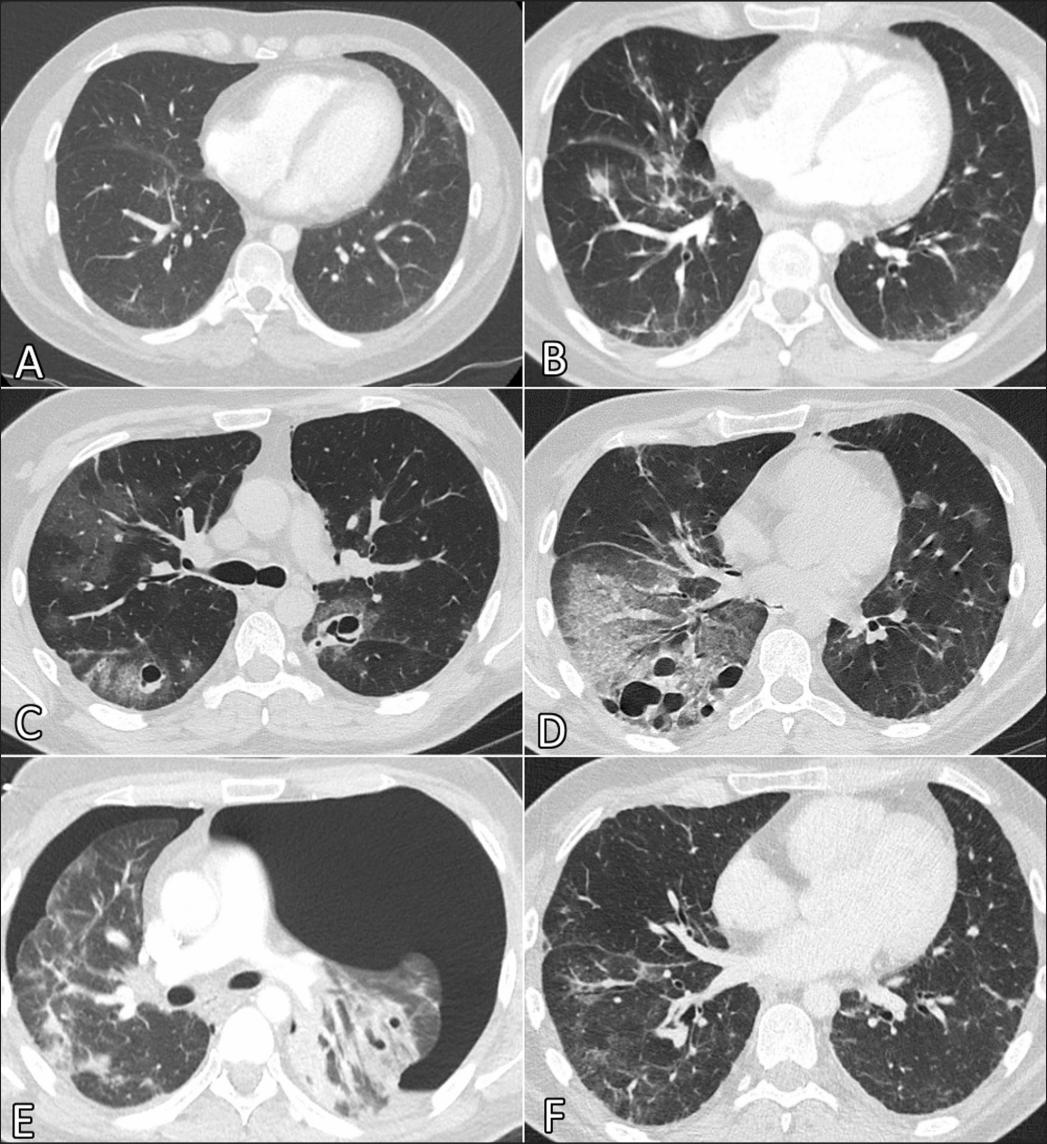
Progression of pulmonary abnormalities on chest CT.

A 29-year-old male with stage IIA mixed germ cell tumor was treated with orchiectomy and adjuvant Bleomycin, Etoposide and Cisplatinum chemotherapy. Due to a decrease in diffusion capacity (DLCO = 71%) after 2 cycli, bleomycin toxicity was suspected and discontinued. Chest CT (Figure 1a) showed discrete reticulations and ground-glass opacities (GGOs) in the lower lobes.

A follow-up chest CT two months later showed more extensive reticulations, septal thickening, consolidation and GGOs (Figure 1b). The diffusion capacity had only worsened slightly but there was no dyspnea. Ten days later the patient was admitted to the emergency department with haemoptysis and dyspnea. Chest CT revealed multiple pneumatoceles of varying sizes, a pneumomediastinum and an increase of air-space opacities and reticulations (Figure 1c–d). Necrotizing pneumonitis was suspected, and antibiotic therapy was started; however, bronchoalveolar lavage (BAL) was negative for infection or malignancy and symptoms spontaneously improved.

A month later the patient returned to the emergency department for acute dyspnea and haemoptysis. Chest CT revealed a bilateral pneumothorax and left-sided tension pneumothorax (Figure 1e). The pneumatoceles had mostly disappeared. After chest tube placement, a VATS talcage and wedge biopsy was performed, and the patient could leave the hospital in good condition. One month later there was complete regression of the consolidation and pneumatoceles on chest CT; however, some GGOs and evidence of fibrosis persisted (Figure 1f).

**Figure 2:**
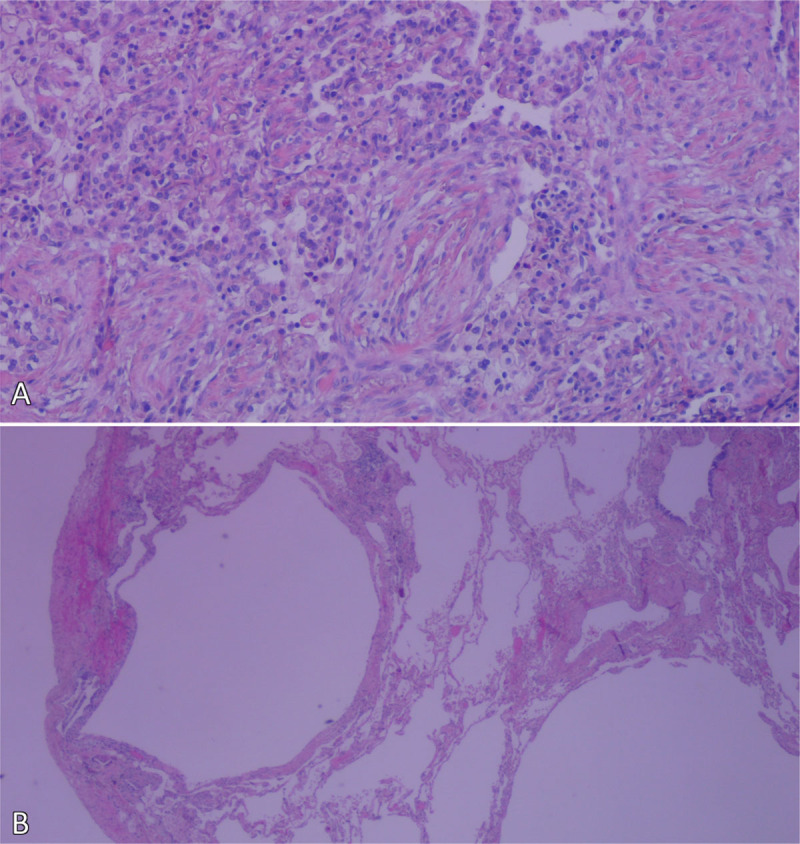
H&E stain showing the **(A)** fibroblastic plugs (Masson bodies) and **(B)** subpleural blebs.

Histopathological examination showed intra-alveolar and intra-bronchial fibroblastic plugs compatible with organizing pneumonia, as well as fibrotic subpleural thickening and intrapleural blebs (Figure 2).

## Comments

Bleomycin is a common cause for drug-induced pulmonary toxicity, with a broad presentation including diffuse alveolar damage, hypersensitivity pneumonitis and organizing pneumonia (OP) [1]. OP is a reparative reaction to lung injury, where the alveoli and bronchioles are filled with buds of granulation tissue consisting of fibroblasts, fibrin and leukocytes, so-called Masson bodies. While primarily an air-space process, there can be variable amounts of interstitial inflammation [4].

In our case there were clinical signs of pulmonary bleomycin toxicity (reduced diffusion capacity) while chest CT initially showed only discrete interstitial changes consisting of peripheral reticulations (Figure 1a). Some OP cases develop more interstitial than air-space inflammation in the initial phase, characterised by reticulations, septal thickening and a paucity of alveolar opacities, resembling nonspecific interstitial pneumonia (NSIP). Only later there is migration of fibroblasts from the interstitial space into the air space, resulting in the typical consolidations and GGOs [4]. Indeed, these opacities only appeared two months after the reticulations (Figure 1b–d).

Air leak syndrome (ALS) consists of a pneumothorax, subcutaneous emphysema and pneumomediastinum and is a potential complication of multiple lung diseases. It is, however, very rare in OP, only described in case reports [2,3,5,6,7,8]. There have been only two previous case reports where bleomycin-induced OP has caused ALS [3,5]. The first sign of ALS was the appearance of pneumatoceles, predominantly in the areas with most disease activity. While the exact mechanism of their formation is not clear, histopathological studies suggest a partial obstruction of the bronchioles by Masson bodies, creating a check-valve mechanism. Overpressure in the affected alveoli causes overinflation and formation of pneumatoceles. The intrapleural blebs on histopathological examination support this hypothesis, and rupture of a pneumatocele is most likely the cause of the pneumothorax. Air from a ruptured cyst can also dissect along the bronchovascular sheath towards the mediastinum, causing a pneumomediastinum and subcutaneous emphysema [2,3,6,7,8]. After the diagnosis of organizing pneumonia was confirmed, corticosteroid therapy was initiated with a good clinical response.
